# The prognostic value of estrogen receptor beta and proline-, glutamic acid- and leucine-rich protein 1 (PELP1) expression in ovarian cancer

**DOI:** 10.1186/1471-2407-13-115

**Published:** 2013-03-14

**Authors:** Stefanie Aust, Peter Horak, Dietmar Pils, Sophie Pils, Christoph Grimm, Reinhard Horvat, Dan Tong, Bernd Schmid, Paul Speiser, Alexander Reinthaller, Stephan Polterauer

**Affiliations:** 1 Department of Gynaecology and Gynaecological Oncology, Comprehensive Cancer Center, Medical University of Vienna, Vienna, 1090, Austria; 2 Department of Internal Medicine, Comprehensive Cancer Center, Medical University of Vienna, Vienna, 1090, Austria; 3 Department of Pathology, Medical University of Vienna, Vienna, 1090, Austria; 4 Department of Gynaecology and Gynaecological Oncology, Hietzing Hospital Vienna, Vienna, 1130, Austria

**Keywords:** PELP1, Estrogen receptor alpha, Estrogen receptor beta, Immunohistochemistry, Prognosis, Ovarian cancer

## Abstract

**Background:**

Proline-, glutamic acid-, and leucine-rich protein 1 (PELP1), a coregulator of the estrogen receptors (ERs) alpha and beta, is a potential proto-oncogene in hormone dependent gynecological malignancies. To better understand the role of PELP1 in epithelial ovarian cancer (EOC), the protein expression and prognostic significance of PELP1 was evaluated together with ERalpha and ERbeta in EOC tissues.

**Methods:**

The expression of PELP1, ERalpha, and ERbeta was characterized in tumor tissues of 63 EOC patients. The prognostic value was calculated performing log-rank tests and multivariate Cox-Regression analysis. In a second step, validation analysis in an independent set of 86 serous EOC patients was performed.

**Results:**

Nuclear PELP1 expression was present in 76.2% of the samples. Prevalence of PELP1 expression in mucinous tumors was significantly lower (37.5%) compared to serous (85.7%) and endometrioid tumors (86.7%). A significant association between PELP1 expression and nuclear ERbeta staining was found (p=0.01). Positive PELP1 expression was associated with better disease-free survival (DFS) (p=0.004) and overall survival (OS) (p=0.04). The combined expression of ERbeta+/PELP1+ revealed an independent association with better DFS (HR 0.3 [0.1-0.7], p=0.004) and OS (HR 0.3 [0.1-0.7], p=0.005). In the validation set, the combined expression of ERbeta+/PELP1+ was not associated with DFS (HR 0.7 [0.4-1.3], p=0.3) and OS (HR 0.7 [0.3-1.4], p=0.3).

**Conclusion:**

Positive immunohistochemical staining for the ER coregulator PELP1, alone and in combination with ERbeta, might be of prognostic relevance in EOC.

## Background

Despite increasing knowledge in the etiology and treatment of epithelial ovarian cancer (EOC), ovarian cancer still accounts for more death women than any other gynecological malignancy [1]. As ovarian cancer is an endocrine-related cancer, the role of estrogen in the complex network of ovarian cancer pathogenesis, as well as the role of estrogen receptors (ERs) as prognostic markers, are being intensively discussed [2,3].

In ovarian cancer cells, estrogen is biologically active by binding to the nuclear receptors (NRs) ERalpha and ERbeta. NR signaling leads to a translocation of the ER to the nucleus, followed by a binding to the target gene promoters and consequently to a specific gene transcription [4]. Inhibition or enhancement of transcription is achieved with the help of ligand-regulated coactivators and corepressors, stabilizing and enhancing or repressing transcription [5]. Deregulation of such NR-coactivators - or coregulators - in ovarian cancer cells is likely to interact with proliferation and survival of cancer cells. The increasing interest in coregulators as mediators of NR function has revealed proline-, glutamic acid-, and leucine-rich protein 1 (PELP) 1, also named “modulator of nongenomic actions of estrogen receptor” (MNAR), as mediator of not only genomic but also non-genomic actions of ERs and as potential proto-oncogene in hormonal cancers [6].

PELP1 interacts with various NRs, including ERs, androgen receptors, glucocorticoid receptors and progesterone receptors [7]. In cancer cells, PELP1 seems to effect ER-mediated gene expression, subsequently influencing cell proliferation and differentiation [8]. In endometrial cancer, PELP1 functionally interacts with both, ERalpha and ERbeta, and enhances their transcriptional response [9]. Furthermore, PELP1 has been associated with increased cell motility and invasion in cancer cells [10]. Elevated PELP1 expression has also been associated with poor outcome in ER positive/luminal-like breast cancer tissue [11].

In ovarian cancer cell line models and in nude mouse models elevated PELP1 expression leads to increased cell migration, metastasis and tumor progression [12]. In human ovarian cancer tissue, only one study has focused on the role of PELP1 protein expression [13]. PELP1 has been described to be overexpressed in 60% of ovarian cancers and to be deregulated in several subtypes of ovarian tumors [13]. It has been proposed, that during ovarian cancer progression, modification of PELP1 expression might occur. According to these findings, the prognostic relevance of PELP1 in ovarian cancer needs to be further elucidated. Additionally, the prognostic importance of ERalpha and ERbeta in ovarian cancer is still discussed controversially, despite their importance in breast and endometrial cancer [14-18].

The major aim of this study was to further define the role of PELP1 in human ovarian cancer and to evaluate the prognostic role of PELP1, ERalpha and ERbeta in EOC patients receiving platinum/taxane-based chemotherapy.

## Methods

### Study population

Paraffin embedded ovarian tumor tissue from primary surgery was used from 63 patients with EOC FIGO stage I-IV, receiving cytoreductive surgery at the Department of General Gynaecology and Gynaecological Oncology, Medical University of Vienna, Austria, between 1996 and 2001. In a second step, a validation set was used comprising 86 EOC patients of only serous histology, receiving cytoreductive surgery at the Medical University of Vienna, Austria, between 2004 and 2010. An informed consent according to the criteria of the Medical University of Vienna was obtained from all patients. Approval for this study was obtained by the institutional review board of the Medical University of Vienna (IRB-no.:266/2010). Patients were treated according to standards of the present institution with upfront surgery and adjuvant platinum–based chemotherapy. Surgical staging according to FIGO guidelines was performed, including hysterectomy, bilateral salpingo-oophorectomy, pelvic and/or paraaortic lymphadenectomy, appendectomy, omentectomy and cytoreductive procedure in order to resect all gross tumor. All patients with tumor stages FIGO Ic to III and all patients with clear cell carcinoma received a platinum-based chemotherapy. Patients wishing to preserve fertility and tumor stage FIGO Ia were treated with conservative surgery (unilateral salpingo-oophorectomy) and full surgical staging including washings, omentectomy, appendectomy, node biopsies and a thorough abdominal exploration with biopsies of all suspicious areas. Residual tumor load was defined as negative, if macroscopically absent. Post-therapeutically all patients were followed up four times annually, including pelvic examination, abdominal ultrasound examination, and serum tumor marker evaluation. Overall survival was the time interval between diagnosis and death. Overall observation time was the time interval between diagnosis and last contact, defined as death from the disease or last follow-up. Recurrent disease was defined as at least a twofold increase in the nadir serum CA-125 level after first-line chemotherapy or radiological diagnosis. Patients without recurrence, cancer progression or death were censored at the time of last follow-up. Experienced gynecological oncologists and an experienced pathologist performed the clinical and histopathological evaluation and the evaluation of response to first-line treatment.

### Ovarian tissue microarray and Immunohistochemistry

Paraffin embedded tissue-blocks were processed using standardized procedures. Tissue microarrays (TMAs) was assembled by taking three core needle ‘biopsies’ from defined tumor regions in the preexisting paraffin-embedded tissue blocks, using techniques and an apparatus developed by Beecher Instruments Inc., Micro-Array Technology (Sun Prairie, WI, USA). Separate TMAs were constructed for the test and validation set. Immunohistochemistry (IHC) procedures were performed at room temperature. Samples were deparaffinized, rehydrated and treated with 3% H2O2 for 10 minutes to quench endogenous peroxidase. For ERbeta antigen heat retrieval was performed using heat-induced epitope retrieval in DakoCytomation Target Retrieval Solution (No. S1700, DAKO, Denmark), and for ERalpha and PELP1 heat-induced epitope retrieval was performed using citrate buffer (Citra-BioGenex No. HK 087-5K). The sections were incubated at 4°C overnight with primary antibodies (ERalpha, 1:50, clone 1D5, mouse IgG1, Dako, Denmark; ERbeta1, 1:20, clone PPG5/10, mouse IgG2a, Dako, Denmark; PELP1, 1:500, polyclonal rabbit, No. IHC-00013, Bethyl Laboratories, USA). As positive controls, FFPE sections of ER positive human breast adenocarcinoma were used. Negative control mouse and rabbit isotypes were used as negative controls. Slides were incubated for 25 minutes with a biotinylated secondary antibody (Link, No. K0673, Dako, Denmark), followed by incubation with Streptavidin Peroxidase-HRP (25 Minutes, No. K 0673, Dako, Denmark). The slides were stained with diamino-benzidine (DAB Chromogen 1:50 in DAB Substrate Buffer, K0673, Dako, Denmark) for 2 minutes. For counterstaining the slides were dipped into hematoxylin for 25 seconds. Intra-nuclear distribution of PELP1 was determined by immunofluorescence staining. The fluorescence labeled secondary antibody, goat anti-rabbit (1:5000; Invitrogen, AlexaFluor® 488 fragment of goat anti-rabbit IgG (H+L)) was used besides DAPI for nuclear counterstaining.

### Data analysis and Statistics

The intensity patterns and nuclear positivity of staining were analyzed by two independent co-workers, including a gynecological pathologist (RH), applying a semi-quantitative scale of ImmunoReactive Score (IRS) calculated by multiplication of the number of positively labeled cells (4 percentage groups) with the intensity of the staining reaction (3 grades) [19]. The intensity of reaction was scored as negative (intensity IRS 0–2, no reaction, and IRS 3–4 showing a very weak reaction of staining with <10% positive cells) or positive (IRS 6–8 with a moderate reaction and 10 to 50% positive cells; IRS 9–12 with a strong reaction and 51% to 80% positive cells). Statistical analyses were performed using SPSS software version 19 (IBM Corporation, Armonk, New York, United States). *P*-values <0.05 were considered statistically significant. Associations between ERalpha-expression, ERbeta-expression, PELP1-expression, and clinicopathological parameters were calculated using Pearson’s *χ*^2^ or Fisher’s exact tests as appropriate. Impact of the potential new prognostic factors on disease-free survival (DFS) and overall survival (OS) was determined by univariate and multiple Cox proportional-Hazards model analyses, whereby in the test set, only the patients receiving chemotherapy according to standardized protocols were included in these analyses (n=50). In the validation set, only patients receiving chemotherapy were included. Survival analyses were performed for all three parameters and the combination pattern ERbeta+/PELP1+ versus ERbeta+/Pelp1-, ERbeta-/PELP1+ and ERbeta-/Pelp1- tumors as well as for the combination pattern ERalpha+/PELP1+ versus ERalpha+/Pelp1-, ERalpha-/PELP1+ and ERalpha-/Pelp1- tumors.

## Results

### Study population

The characteristics of the patients included in the study show a typical heterogeneous ovarian cancer population and are depicted in Table 1. Mean age of the EOC patients at time of cytoreductive surgery was 58.3 years (±13.9years). The mean observation period was 57.8 months. Within the observation period, 36 patients died (57.1%) and 36 patients (57.1%) experienced tumor recurrence. 50 patients (79.4%) received carboplatin-paclitaxel based standard chemotherapy. Out of these 50 patients, 46 women (73.0%) showed initial response to chemotherapy and a total of 34 women died (68.0%). The characteristics of the validation set are likewise presented in Table 1. In this validation set the mean observation time was 51.3 months and a total of 45 (52.3%) patients died.

**Table 1:** **Patients**’ **characteristics**

	**Screening set (n** = **63)**	**Validation set (n** = **86)**
**Characteristics**	**n** (%)	**n** (%)
**Histology**		
Serous	28 (44.4)	86 (100.0)
Non-serous	35 (55.6) ^1^	-
**FIGO**		
I	19 (30.2)	6 (7.0)
II	6 (9.5)	4 (4.7)
III	32 (50.8)	61 (70.9)
IV	6 (9.5)	15 (17.4)
**Grade**		
Grade 1	10 (15.9)	6 (7.0)
Grade 2	20 (31.7)	19 (22.1)
Grade 3	33 (52.4)	61 (70.9)
**Residual tumor**		
no	35 (55.6)	37 (43.0)
> 0 cm	28 (44.4)	49 (57.0)
**ERα**		
positive	13 (20.6)	27 (31.4)
negative	49 (77.8)	55 (63.9)
missing	1 (1.5)	4 (4.7)
**ERbeta**		
positive	45 (71.4)	14 (16.3)
negative	15 (23.8)	68 (79.0)
missing	3 (4.7)	4 (4.7)
**PELP1**		
positive	48 (76.2)	21 (24.4)
negative	12 (19.0)	61 (70.9)
missing	3 (4.7)	4 (4.7)

^*1*^*Endometrioid*: *n*= *16*; *Mucinous*: *n*= *9*; *Undifferentiated carcinoma*: *n*= *7*; *Clear cell carcinoma*: *n*= *3*;

### Distribution of PELP1 expression

**Figure 1:**
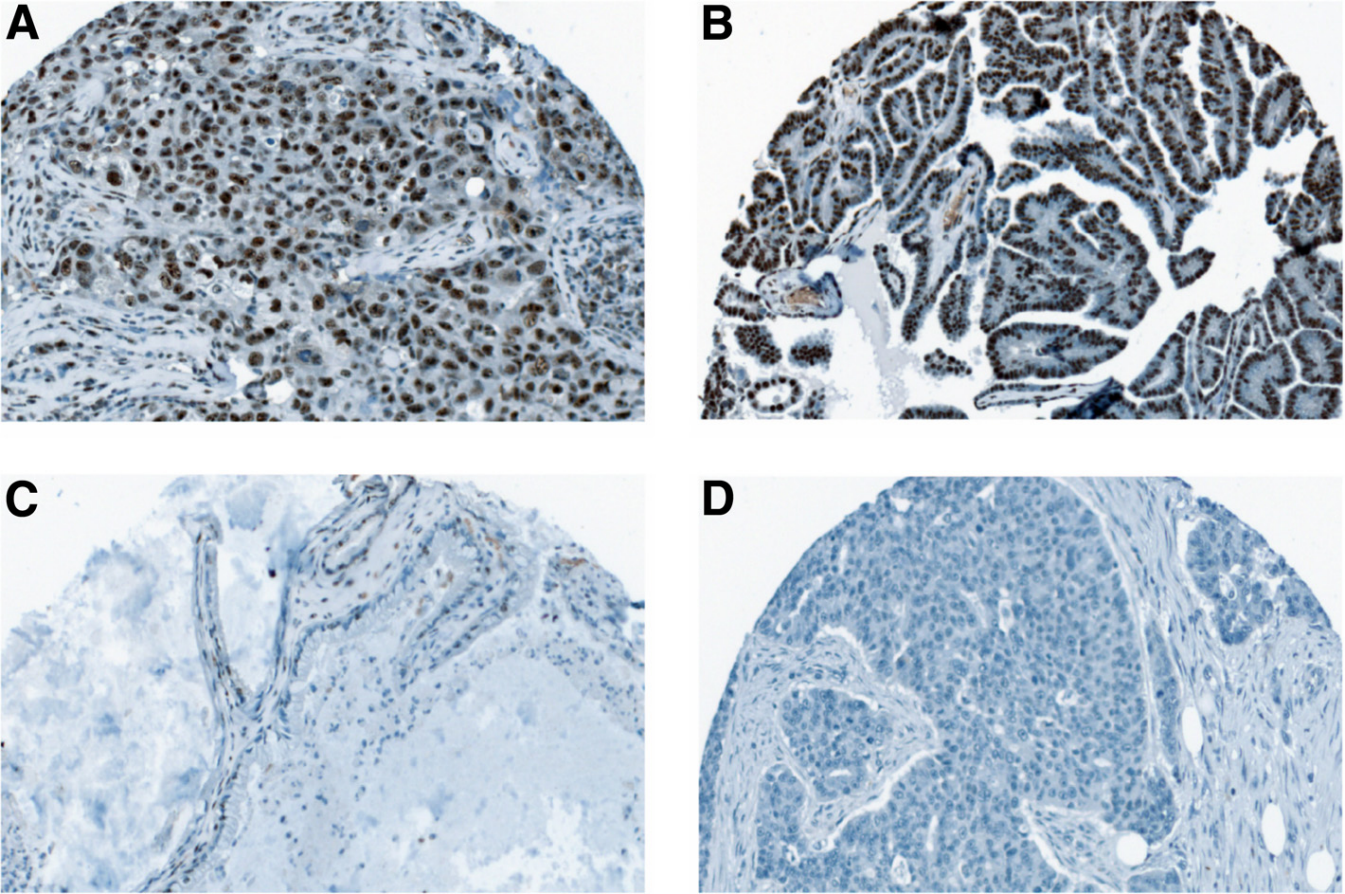
**Representative immunohistochemical examples of PELP1 staining.** In (**A**) PELP1 positive low differentiated epithelial ovarian cancer, (**B**) PELP1 positive high differentiated epithelial ovarian cancer, (**C**) mucinous ovarian cancer with nearly complete absence of nuclear PELP1 staining and (**D**) PELP1 negative low differentiated epithelial ovarian cancer is shown. Pictures were taken using TissueFAXS (TissueGnostics; Vienna, Austria; magnification x200).

**Figure 2:**
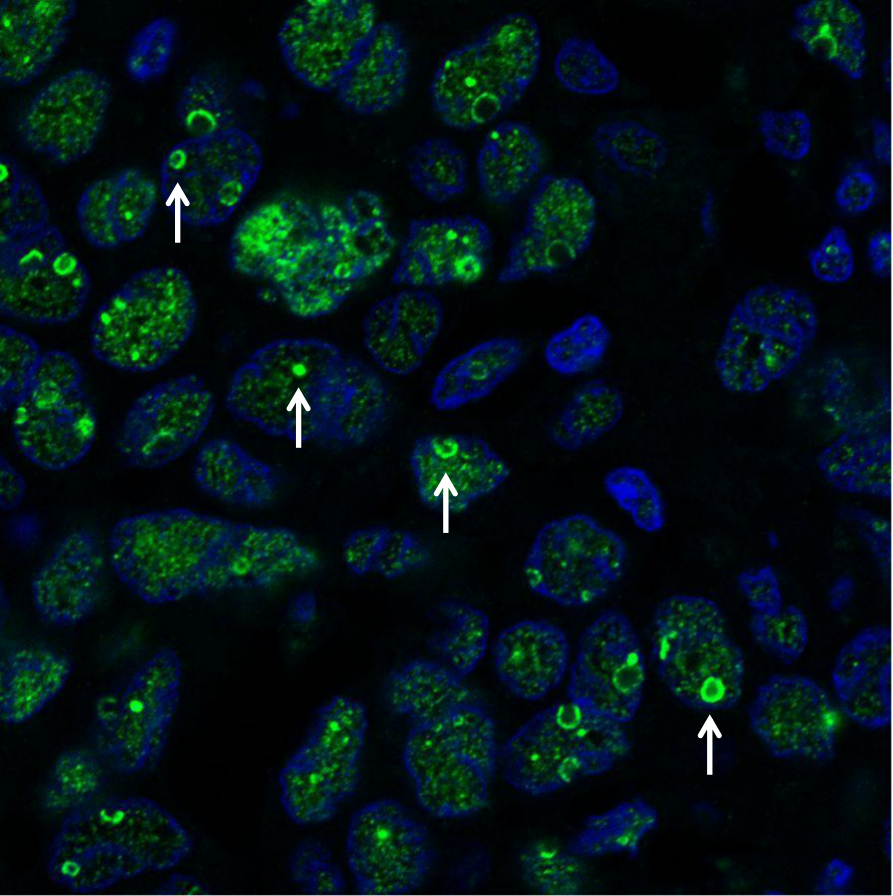
**Intra**-**nuclear distribution of PELP1 in a serous EOC sample.** An intense PELP1 staining (green) can be observed within the nucleolar compartments (white arrows). DAPI (blue) was used for nuclear counterstaining (Pictures were taken with the confocal microscope LSM700).

Of the 63 cancer tissues, 48 (76.2%) of patients were found to express nuclear staining of PELP1. Out of the 48 patients classified as PELP1 positive, a moderate PELP1 expression was found in 24 (38.1%) tissues and a strong expression was observed in 23 (36.5%) of the samples. Representative immunohistochemical examples of PELP1 positive and negative stainings in ovarian cancer tissue are shown in Figure 1A-D. Using immunofluorescence, intra-nuclear distribution of PELP1 was analyzed. As depicted in Figure 2, an intensive PELP1 staining within the nucleoli of the tumor cells can be observed. Further examination of histological subtypes revealed expression of PELP1 in all included subtypes of ovarian cancer. All undifferentiated carcinomas were PELP1 positive, as was the majority of serous and endometrioid tumors (85.7% and 86.7%, respectively), whereas the majority of mucinous tumors (62.5%) showed no nuclear PELP1 expression (p=0.02, Fisher’s exact). No significant difference of PELP1 expression regarding age (<55 years *vs* > 55 years), FIGO stage, grade or residual tumor load after cytoreductive surgery could be observed.

### Distribution of ERalpha and ERbeta expression

Nuclear staining of ERalpha and ERbeta was positive in 13 (20.6%) and in 45 (71.4%) ovarian cancer patients, respectively. Examination of ERalpha and ERbeta expression among the different histological tumor types revealed no significant differences (p=0.4 and p=0.2, respectively; Fisher’s exact). ERbeta expression could be observed in all subtypes. A total of 85.2% serous, 44.4% mucinous, 73.3% endometrioid, 71.4% undifferentiated and both clear cell carcinomas (100%) were ERbeta expression positive. A higher percentage of ERalpha expressing tumors was observed among serous (29.6%) compared to endometrioid (18.8%) and undifferentiated carcinomas (28.6%). No ERalpha expression was seen in the clear cell carcinomas and the mucinous ovarian carcinomas. Regarding age (≤ 55 years *vs* > 55 years), FIGO-stage, grade or residual tumor load after debulking surgery, no significant differences were found among ERalpha and/or ERbeta expressing tumors (data not shown).

### Coexpression of PELP1 and ERs

In a next step, we analyzed the coexpression of PELP1 together with ERalpha and ERbeta. A significant association between PELP1 expression and nuclear ERbeta staining was found (p=0.01, Fisher’s exact), whereas no significant association between PELP1 and nuclear ERalpha staining was observed (p=0.3, Fisher’s exact). Expression of PELP1 and both ERs was observed in 10 patients (15.8%).

### Survival analyses

Table 2A shows the association between PELP1, ER-alpha, ERbeta and survival. In a univariate analysis, ovarian cancer patients with PELP1 expressing tumor tissue had a better OS and DFS (p = 0.04, p = 0.004; respectively) compared to patients without PELP1 expression. ERbeta and ERalpha had no significant univariate influence on survival. Interestingly, the coexpression of PELP1 and ERbeta turned out to be the most relevant prognostic factor in univariate survival analysis (DFS: p=0.03, OS: p=0.02). In a univariate analysis, the clinicopathological parameters FIGO-stage (p < 0.001 and p < 0.001) and residual tumor (p < 0.001 and p < 0.001) were associated with DFS and OS, respectively.

**Table 2:** **Survival analysis of ovarian cancer patients including coexpression of PELP1**/**ERbeta in the test set** (**A**) **and validation set** (**B**)

	**Disease**-**free survival**			**Overall survival**		
	**Univariate**^**1**^	**Multivariate**^**2**^		**Univariate**^**1**^	**Multivariate**^**2**^	
	***P***-**Value**	***P***-**Value**	**HR** (**95**%**CI**)	***P***-**Value**	***P***-**Value**	**HR** (**95**%**CI**)
**Patients**’ **age**	0.9	0.09	2.1 (0.9-4.9)	0.9	0.4	1.4 (0.6-3.0)
**Histological type** (serous vs. non-serous)	0.6	0.6	0.8 (0.4-1.8)	0.4	0.9	0.9 (0.5-2.2)
**FIGO stage** (I vs. II vs. III vs. IV)	<**0**.**001**	<**0**.**001**	**4**.**4** (**1**.**9**-**10**.**1**)	<**0**.**001**	**0**.**007**	**3**.**2** (**1**.**4**-**7**.**6**)
**Residual tumor**(no vs. yes)	<**0**.**001**	0.9	0.9 (0.4-2.6)	<**0**.**001**	0.4	1.6 (0.6-4.3)
**ERalpha** (negative vs. positive)	0.8	-	-	0.9	-	-
**ERbeta** (negative vs. positive)	0.3	-	-	0.2	-	-
**PELP1** (negative vs. positive)	**0**.**004**	-	-	**0**.**04**	-	-
**PELP1**/**ERbeta** (ERbeta+/PELP1+ vs. others)	**0**.**03**	**0**.**004**	**0**.**3** (**0**.**1**-**0**.**7**)	**0**.**02**	**0**.**005**	**0**.**3** (**0**.**1**-**0**.**7**)
	**Disease**-**free survival**			**Overall survival**		
	**Univariate**^**1**^	**Multivariate**^**2**^		**Univariate**^**1**^	**Multivariate**^**2**^	
	***P***-**Value**	***P***-**Value**	**HR** (**95**%**CI**)	***P***-**Value**	***P***-**Value**	**HR** (**95**%**CI**)
**Patients**’ **age**	0.09	0.4	1.3 (0.7-2.2)	**0**.**005**	**0**.**04**	**2**.**4** (**1**.**1**-**5**.**3**)
**FIGO stage** (I vs. II vs. III vs. IV)	**0**.**001**	**0**.**02**	1.8 (1.1-3.0)	**0**.**001**	**0**.**005**	**2**.**5** (**1**.**3**-**4**.**7**)
**Residual tumor** (no vs. yes)	**0**.**003**	0.1	1.6 (0.9-1.8)	**0**.**02**	0.3	1.4 (0.7-2.7)
**ERalpha** (negative vs. positive)	0.2	-	-	0.6	-	-
**ERbeta** (negative vs. positive)	0.7	-	-	0.2	-	-
**PELP1** (negative vs. positive)	0.8	-	-	0.9	-	-
**PELP1**/**ERbeta** (ERbeta+/PELP1+ vs. others)	0.6	0.3	0.7 (0.4-1.3)	0.9	0.3	0.7 (0.3-1.4)

^1^Log rank test; ^2^multivariate Cox-regression analysis, HR=Hazard Ratio, 95%CI= 95% Confidence Interval.

In a next step, we added the coexpression of PELP1 and ERbeta and all clinicopathological parameters in a Cox proportional-hazards regression model. Multiple Cox regression analysis revealed that ERbeta+/PELP1+ tumors had the strongest independent impact on survival, with a significantly longer DFS (HR 0.3 [0.1-0.7], p = 0.004) and OS (HR 0.3 [0.1-0.7], p = 0.005).

To provide more profound and histotype specific survival data we have additionally performed a validation analysis in a validation set of 86 EOC with only serous histology. In univariate and multivariate analysis, ERbeta+/PELP1+ expression had no significant influence on survival in this validation set of only serous histology. Survival data is presented in Table 2B.

## Discussion

In this study, coexpression of PELP1 and ERbeta was associated with a better prognosis in patients with EOC. We primarily investigated the expression of PELP1, ERalpha and ERbeta in 63 human EOC tissues. Nuclear PELP1 expression was present in 76.2% and was found in all histological subtypes. To our knowledge, only one study has previously reported on PELP1 expression in human EOC tissue [13], whereby no differences could be observed between the four major types of EOC. Vadlamudi et al. speculated that PELP1modulates rDNA transcription and accelerates cell cycle progression. Using immunofluorescence staining of MCF7 and HeLa cells, they discovered that PELP1 localizes in the nucleolar compartments [6]. Thus we decided to perform immunofluorescence to investigate intranuclear expression of PELP1 in EOC. Our results show, that in serous EOC, an intense nucleolar expression of PELP1 can be observed. Further studies are required to determine PELP1 expression during cell-cycle progression in EOC.

In our study-population, the majority of serous and endometrioid tumor tissues were PELP1 positive, whereas we observed a significantly reduced number of PELP1 expressing mucinous tumors (37.5%). Additionally, none of the mucinous tumors expressed ERalpha. Primary mucinous tumors are comparatively rare and they were cautiously classified in this study [20]. Molecular changes in mucinous tumors have not been studied to the same extend as in serous and endometrioid tumors [21], highlighting the importance to further characterize this histological subtype. Matching our results, a previous study has reported mucinous EOC to be ERalpha negative [22]. Additionally, a significantly higher ER expression was observed in serous (43% positive) versus mucinous (4% positive) EOCs in a large study, including over 700 ovarian cancer patients [14]. Our results show, that mucinous tumors remained ERbeta positive, which is also in accordance with the findings by Lindgren et al. [22].

Our findings reflect relatively low frequencies of ERalpha expression versus ERbeta expression, with 20.6% versus 71.4%, respectively. This is in accordance with a previous immunohistochemical evaluation in EOC, describing a higher ERbeta than ERalpha expression [15]. Interacting proteins have been described for PELP1 and both nuclear hormone receptors [8]. In our study, a similar expression pattern was observed between ERbeta and PELP1, as proven by a significant correlation. This may indicate a functional interaction especially between ERbeta and PELP1 in EOC. In ER transactivation assays, PELP1 seems to potentiate ERbeta mediated transcriptional activity in endometrial cells [9]. To determine the impact of PELP1 on ER transcriptional activity, additional in vitro studies with ovarian cancer cells need to be set up.

The prognostic impact of ER expression on patients’ survival has been discussed in literature over years but still remains controversial. ER expression was described as a favorable marker in a variety of studies [14,15,23]. However, reports on ER as not significantly influencing patients’ survival [17,24] have likewise been published. To determine the association between ER and survival, coregulators interacting with these NRs and often deregulated in hormone-interacting tumors [25] should likewise be taken into account. Our analysis revealed that women with positive PELP1 expression had a better OS and DFS compared to patients without PELP1 expression. We know that the patient number of our test set is a limitation for survival analysis, but in view of the low patient number, the positive impact we could observe for PELP1 in univariate survival analysis is even more interesting. As we have observed a correlation between PELP1 and ERbeta expression, we additionally analyzed the coexpression of ERbeta and PELP1 regarding survival. Besides the clinicopathological factors FIGO stage and residual tumor load after cytoreductive surgery, this coexpression pattern (ERbeta+/PELP1+) turned out to be the most relevant prognostic factor in univariate and multivariate survival analysis, revealing a significantly longer DFS (HR 0.3 [0.1-0.7], p = 0.004) and OS (HR 0.3[0.1-0.7], p = 0.005).

Due to these positive findings we validated the survival data in a validation set. The validation was performed in an independent more homogenous set of 86 EOC patients with only serous histology. Unfortunately we could not reproduce our findings regarding the protective effect of the coexpression of ERbeta and PELP1. Both results are of relevance as little is known about the role of PELP1 in EOC. PELP1 is being discussed as a potentially targetable proto-oncogene in ER positive breast cancer [26], associated with rapid tumor growth in xenograft models and high grade as well as node-positivity in human breast cancer [27]. PELP1 seems to be of importance in breast cancer, due to an involvement in hormone therapy response and resistance [25]. In EOC, hormone therapy has been studied in a limited number of trials, including mainly patients with recurrent or refractory ovarian cancer. To understand not only the prognostic, but also the therapeutic role of ERs in EOC, further studies on coregulators and the mechanisms through which hormones interact with EOC need to be designed.

The findings of our test set remain of importance as Grivas et al. have described PELP1 overexpression in epithelial colorectal cancer cells to be associated with increased OS. Comparable to our results, a correlation between ERbeta and PELP1 was observed, and PELP1 was a positive prognostic marker in the subset of ERbeta positive carcinomas [28].

## Conclusion

Potential prognostic markers for women diagnosed with ovarian cancer are urgently needed. In conclusion, our results show that PELP1 expression, alone and in combination with ERbeta, may be an interesting research target in this cancer entity.

## Competing interests

The authors have no competing interests to declare.

## Authors’ contribution

SA carried out the immunohistochemical analyses and has been involved in the draft of the manuscript. PH has been involved in construction of the validation TMA and data generation. DP carried out the statistical analyses and has been involved in data interpretation. SP has been involved in clinical data generation. StP has been involved in drafting of the manuscript and the clinical data generation. RH carried out the pathological examination of the immunohistochemical stainings. DT carried out the statistical analyses. BS has been involved in the construction of the test TMA and the data generation. PS has been involved in the clinical data generation. AR has been involved in the data interpretation and participated in the design of the study. CG participated in the design of the study and helped to draft the manuscript. All authors read and approved the final manuscript.

## Pre-publication history

The pre-publication history for this paper can be accessed here:

http://www.biomedcentral.com/1471-2407/13/115/prepub

## References

[B1] Jemal A Siegel R Ward E Murray T Xu J Thun MJ CA Cancer J Clin 2007 57 1 43 66 10.3322/canjclin.57.1.43 17237035

[B2] Gallo D Ferlini C Scambia G The epithelial-mesenchymal transition and the estrogen-signaling in ovarian cancer Curr Drug Targets 2010 11 4 474 481 10.2174/138945010790980385 20015012

[B3] Park SH Cheung LW Wong AS Leung PC Estrogen regulates Snail and Slug in the down-regulation of E-cadherin and induces metastatic potential of ovarian cancer cells through estrogen receptor alpha Mol Endocrinol 2008 22 9 2085 2098 10.1210/me.2007-0512 18550773 PMC5419456

[B4] Horwitz KB Jackson TA Bain DL Richer JK Takimoto GS Tung L Nuclear receptor coactivators and corepressors Mol Endocrinol 1996 10 10 1167 1177 10.1210/me.10.10.1167 9121485

[B5] O'Malley BW Coregulators: from whence came these "master genes" Mol Endocrinol 2007 21 5 1009 1013 10.1210/me.2007-0012 17284664

[B6] Gonugunta VK Nair BC Rajhans R Sareddy GR Nair SS Vadlamudi RK Regulation of rDNA transcription by proto-oncogene PELP1 PLoS One 2011 6 6 e21095 10.1371/journal.pone.0021095 21695158 PMC3113909

[B7] Vadlamudi RK Kumar R Functional and biological properties of the nuclear receptor coregulator PELP1/MNAR Nucl Recept Signal 2007 5 e004 17525794 10.1621/nrs.05004 PMC1876599

[B8] Wong CW McNally C Nickbarg E Komm BS Cheskis BJ Estrogen receptor-interacting protein that modulates its nongenomic activity-crosstalk with Src/Erk phosphorylation cascade Proc Natl Acad Sci U S A 2002 99 23 14783 14788 10.1073/pnas.192569699 12415108 PMC137496

[B9] Vadlamudi RK Balasenthil S Broaddus RR Gustafsson JA Kumar R Deregulation of estrogen receptor coactivator proline-, glutamic acid-, and leucine-rich protein-1/modulator of nongenomic activity of estrogen receptor in human endometrial tumors J Clin Endocrinol Metab 2004 89 12 6130 6138 10.1210/jc.2004-0909 15579769 PMC1262662

[B10] Chakravarty D Nair SS Santhamma B Nair BC Wang L Bandyopadhyay A Agyin JK Brann D Sun LZ Yeh IT Extranuclear functions of ER impact invasive migration and metastasis by breast cancer cells Cancer Res 2010 70 10 4092 4101 10.1158/0008-5472.CAN-09-3834 20460518 PMC2889925

[B11] Habashy HO Powe DG Rakha EA Ball G Macmillan RD Green AR Ellis IO The prognostic significance of PELP1 expression in invasive breast cancer with emphasis on the ER-positive luminal-like subtype Breast Cancer Res Treat 2010 120 3 603 612 10.1007/s10549-009-0419-9 19495959

[B12] Chakravarty D Roy SS Babu CR Dandamudi R Curiel TJ Vivas-Mejia P Lopez-Berestein G Sood AK Vadlamudi RK Therapeutic targeting of PELP1 prevents ovarian cancer growth and metastasis Clin Cancer Res 2011 17 8 2250 2259 10.1158/1078-0432.CCR-10-2718 21421858 PMC3731129

[B13] Dimple C Nair SS Rajhans R Pitcheswara PR Liu J Balasenthil S Le XF Burow ME Auersperg N Tekmal RR Role of PELP1/MNAR signaling in ovarian tumorigenesis Cancer Res 2008 68 12 4902 4909 10.1158/0008-5472.CAN-07-5698 18559538

[B14] Hogdall EV Christensen L Hogdall CK Blaakaer J Gayther S Jacobs IJ Christensen IJ Kjaer SK Prognostic value of estrogen receptor and progesterone receptor tumor expression in Danish ovarian cancer patients: from the 'MALOVA' ovarian cancer study Oncol Rep 2007 18 5 1051 1059 17914554

[B15] Burges A Bruning A Dannenmann C Blankenstein T Jeschke U Shabani N Friese K Mylonas I Prognostic significance of estrogen receptor alpha and beta expression in human serous carcinomas of the ovary Arch Gynecol Obstet 2010 281 3 511 517 10.1007/s00404-009-1185-y 19639330

[B16] Arias-Pulido H Smith HO Joste NE Bocklage T Qualls CR Chavez A Prossnitz ER Verschraegen CF Estrogen and progesterone receptor status and outcome in epithelial ovarian cancers and low malignant potential tumors Gynecol Oncol 2009 114 3 480 485 10.1016/j.ygyno.2009.05.045 19560192 PMC2756056

[B17] Lee P Rosen DG Zhu C Silva EG Liu J Expression of progesterone receptor is a favorable prognostic marker in ovarian cancer Gynecol Oncol 2005 96 3 671 677 10.1016/j.ygyno.2004.11.010 15721410

[B18] Munstedt K Steen J Knauf AG Buch T von Georgi R Franke FE Steroid hormone receptors and long term survival in invasive ovarian cancer Cancer 2000 89 8 1783 1791 10.1002/1097-0142(20001015)89:8<1783::AID-CNCR19>3.0.CO;2-D 11042574

[B19] Remmele W Stegner HE: [Recommendation for uniform definition of an immunoreactive score (IRS) for immunohistochemical estrogen receptor detection (ER-ICA) in breast cancer tissue] Pathologe 1987 8 3 138 140 3303008

[B20] Hart WR Mucinous tumors of the ovary: a review Int J Gynecol Pathol 2005 24 1 4 25 15626914

[B21] Cho KR Shih Ie M: Ovarian cancer Annu Rev Pathol 2009 4 287 313 10.1146/annurev.pathol.4.110807.092246 18842102 PMC2679364

[B22] Lindgren PR Cajander S Backstrom T Gustafsson JA Makela S Olofsson JI Estrogen and progesterone receptors in ovarian epithelial tumors Mol Cell Endocrinol 2004 221 1–2 97 104 15223136 10.1016/j.mce.2004.02.020

[B23] Halon A Materna V Drag-Zalesinska M Nowak-Markwitz E Gansukh T Donizy P Spaczynski M Zabel M Dietel M Lage H Estrogen receptor alpha expression in ovarian cancer predicts longer overall survival Pathol Oncol Res 2011 17 3 511 518 10.1007/s12253-010-9340-0 21207255 PMC3158974

[B24] Tangjitgamol S Manusirivithaya S Khunnarong J Jesadapatarakul S Tanwanich S Expressions of estrogen and progesterone receptors in epithelial ovarian cancer: a clinicopathologic study Int J Gynecol Cancer 2009 19 4 620 627 10.1111/IGC.0b013e3181a44b62 19509560

[B25] Chakravarty D Tekmal RR Vadlamudi RK PELP1: A novel therapeutic target for hormonal cancers IUBMB Life 2010 62 3 162 169 20014005 10.1002/iub.287 PMC2997573

[B26] Cortez V Mann M Tekmal S Suzuki T Miyata N Rodriguez-Aguayo C Lopez-Berestein G Sood AK Vadlamudi RK Targeting the PELP1-KDM1 axis as a potential therapeutic strategy for breast cancer Breast Cancer Res 2012 14 4 R108 10.1186/bcr3229 22812534 PMC3680946

[B27] Rajhans R Nair S Holden AH Kumar R Tekmal RR Vadlamudi RK Oncogenic potential of the nuclear receptor coregulator proline-, glutamic acid-, leucine-rich protein 1/modulator of the nongenomic actions of the estrogen receptor Cancer Res 2007 67 11 5505 5512 10.1158/0008-5472.CAN-06-3647 17545633 PMC2774841

[B28] Grivas PD Tzelepi V Sotiropoulou-Bonikou G Kefalopoulou Z Papavassiliou AG Kalofonos H Expression of ERalpha, ERbeta and co-regulator PELP1/MNAR in colorectal cancer: prognostic significance and clinicopathologic correlations Cell Oncol 2009 31 3 235 247 19478391 10.3233/CLO-2009-0467 PMC4618984

